# Antidepressant Utilization and Suicide in Europe: An Ecological Multi-National Study

**DOI:** 10.1371/journal.pone.0066455

**Published:** 2013-06-19

**Authors:** Ricardo Gusmão, Sónia Quintão, David McDaid, Ella Arensman, Chantal Van Audenhove, Claire Coffey, Airi Värnik, Peeter Värnik, James Coyne, Ulrich Hegerl

**Affiliations:** 1 CEDOC, Departamento de Saúde Mental, Faculdade de Ciências Médicas, Universidade Nova de Lisboa, Lisboa, Portugal; 2 Departamento de Psiquiatria e Saúde Mental, Centro Hospitalar de Lisboa Ocidental, Lisboa, Portugal; 3 Personal Social Services Research Unit, LSE Health and Social Care, London School of Economics and Political Science, London, United Kingdom; 4 National Suicide Research Foundation, Cork, Ireland; 5 Katholieke Universiteit Leuven, LUCAS, Leuven, Belgium; 6 Estonian-Swedish Mental Health and Suicidology Institute (ERSI), Tallinn, Estonia; 7 Perelman School of Medicine of the University of Pennsylvania, Philadelphia, Pennsylvania, United States of America; 8 Health Psychology Section, Department of Health Sciences, University Medical Center, University of Groningen, Groningen, The Netherlands; 9 Department of Psychiatry, University of Leipzig, Leipzig, Germany; Nathan Kline Institute for Psychiatric Research and New York School of Medicine, United States of America

## Abstract

**Background:**

Research concerning the association between use of antidepressants and incidence of suicide has yielded inconsistent results and is the subject of considerable controversy. The first aim is to describe trends in the use of antidepressants and rates of suicide in Europe, adjusted for gross domestic product, alcohol consumption, unemployment, and divorce. The second aim is to explore if any observed reduction in the rate of suicide in different European countries preceded the trend for increased use of antidepressants.

**Methods:**

Data were obtained for 29 European countries between 1980 and 2009. Pearson correlations were used to explore the direction and magnitude of associations. Generalized linear mixed models and Poisson regression distribution were used to clarify the effects of antidepressants on suicide rates, while an autoregressive adjusted model was used to test the interaction between antidepressant utilization and suicide over two time periods: 1980–1994 and 1995–2009.

**Findings:**

An inverse correlation was observed in all countries between recorded Standardised Death Rate (SDR) for suicide and antidepressant Defined Daily Dosage (DDD), with the exception of Portugal. Variability was marked in the association between suicide and alcohol, unemployment and divorce, with countries depicting either a positive or a negative correlation with the SDR for suicide. Every unit increase in DDD of an antidepressant per 1000 people per day, adjusted for these confounding factors, reduces the SDR by 0.088. The correlation between DDD and suicide related SDR was negative in both time periods considered, albeit more pronounced between 1980 and 1994.

**Conclusions:**

Suicide rates have tended to decrease more in European countries where there has been a greater increase in the use of antidepressants. These findings underline the importance of the appropriate use of antidepressants as part of routine care for people diagnosed with depression, therefore reducing the risk of suicide.

## Introduction

Antidepressant use has continually increased in most European countries since the advent of selective serotonin reuptake inhibitors (SSRIs). Between 2000 and 2010 rates of use in Europe have continued to increase, with the highest DDD rates seen in Iceland, Denmark and Portugal [1]. Suicide rates vary greatly across the European Economic Area, but between 1980 and 2000 suicide rates fell in all of the EU-15 countries plus Norway, with the exceptions of Ireland and Spain [2]. From 1995 to 2010, the same decrease in suicide rate was observed across the EU-27 countries, with only the exceptions of Malta, Poland and Portugal where increasing trends were present [1]. Despite the onset of the economic crisis, there is no strong evidence that national suicide rates have increased but suicide remains a major public health problem, accounting for 60.000 deaths per year in the EU-27 alone [1].

Suicide is strongly associated with poor mental health, especially mood disorders [3].

Antidepressants are the most common treatment for mood disorders, but effective use of these medications requires administration to patients who have been properly diagnosed and then adequately followed-up [4], [5]. There is a consensus as to the importance of primary care doctors’ education programmes for improving the management of depression with antidepressants in order to reduce the risk of suicide [6]. Furthermore, a number of multi-component suicide prevention programmes emphasise the crucial importance of primary care education programmes to facilitate optimal antidepressant prescribing [7].

However, there are concerns about the efficacy and safety of antidepressants, with some authors suggesting that these medications are at best no better than placebo [8] and others that antidepressants may actually increase the risk of suicidal behaviour, particularly in young people [9]–[11]. In contrast, still other authors contend that there is a bias in these findings and that benefits are in fact greater than risk [12]–[16]. For instance, one meta-analysis of 27 RCT trials examined antidepressant prescribing in children and adolescents to age 18 with a diagnosis of major depressive disorder and showed that benefits appeared to far outweigh a small increased risk of suicidal behaviour [17].

The limited applicability of data from RCTs to public health questions point to the importance of evidence from other types of study design. For instance, analysis of US Veterans Affairs Medical System record data of more than 200.000 adults diagnosed with depression and followed up for at least six months, found statistically significant lower rates of suicide in those treated using any antidepressant [18]. Comparisons among such studies with very different approaches are difficult. Studies vary in basic terminology, definition of outcomes and time periods considered, drugs and other interventions assessed, and statistical methods, leading to seemingly contradictory results. For instance, one review of studies with naturalistic designs had equivocal findings [19]. In contrast, a number of studies of the effects of warnings on the risk of suicide with use of antidepressants on subsequent usage observed an increase in suicide rates in younger people [19]. Furthermore, differing approaches in these studies to controlling for potential confounds are challenging for integration and interpretation. In some settings and contexts, economic development correlates with lower suicide rates [20], [21], while alcohol consumption [22]–[24], divorce both in men and women [25], [26] and unemployment [27], [28] can correlate with higher suicide rates.

Nonetheless, there is potentially an important role for ecological studies, i.e., studies analysing data trends at a population rather than an individual level, to help to inform public health policy. This is advantageous where multiple areas or countries can be examined, in order to control better for region-specific factors that may impact on suicide rates and use of mental health services. The evidence from these studies is, however, also mixed. One review of 19 ecological studies found equivocal evidence for links between suicide and antidepressants, with slightly greater reductions in suicide rates in the 1990s compared to the 1980s, especially when associated with higher initial suicide rates, being a man and older age [29].

Wheeler et al [30] examined changes in country-specific suicide trends in younger people following the introduction of regulatory actions including the use of warnings on antidepressants in a number of countries in 2003 and 2004. They also found the evidence to be equivocal with reductions in the rate of suicide observed in some countries and increases in others, albeit noting weak evidence of an increase in suicide in young women.

Ludwig and Marcotte [31] pooled panel data concerning rates of suicide and the increased use of SSRI’s from the US, Canada, Australia and 24 European countries between 1980 and 2000 and estimated that overall an increase in sales of one pill per capita was found to be associated with a 2.5% decrease in suicide rates for the whole population. However, they acknowledged that this finding was qualified by SSRI sales data having to be imputed prior to 1990 due to a lack of sales data. Nonetheless, their finding suggests that greater utilization of SSRI, particularly for adults, might be a cost effective strategy from a public health perspective, with one suicide averted for every 300,000 pills sold. The same authors also undertook further analysis with panel data covering the same time period for 26 countries, including seven countries from central and Latin America, Japan, Israel, 13 EEA countries, the US, Canada, Australia and New Zealand. The conclusions were much the same, although a more powerful effect was shown with one suicide averted for every 200,000 pills sold. This analysis also noted that there was no evidence in any change in patterns of psychotherapy over the study period [32].

Thus, a number of previous studies have used an ecological approach to look at some actions to help reduce the risk of suicide. Notwithstanding long held arguments on ‘ecological fallacy’ and the danger of misinterpretation of findings of studies gathered using population level data [33], [34], there are at least three reasons for a greater use of this type of study design in respect of suicide research.

First, escalating costs associated with increasing use of antidepressants in many countries suggests the need to examine long term effectiveness of antidepressants both in terms of a reduced prevalence of mood disorders or reduced incidence of suicide. The value of antidepressant treatment at a population-health level has been challenged [35], [36] and remains to be demonstrated. Second, in order to demonstrate statistically in a controlled study that antidepressants produce a preventive effect in respect of the profound but nonetheless relatively rare event of a completed suicide, we would need a sample size of 20.000 people randomly treated with either antidepressants or placebo [37]. This may be difficult to achieve in practice given that suicidal risk tends to be an exclusion criterion in antidepressant trials, naturalistic or experimental. Third, it would be unacceptable for ethical reasons to conduct a randomized controlled trial with suicide as an outcome variable [38], [39].

### Objectives

Given the continued debate on whether evidence of substantial increases in the rate of antidepressant prescription can be translated into improved public health outcomes, and notably reduction in suicide, the present study aims to describe antidepressant utilization and suicide trends in European, largely EU, Member States.

Our first aim was to examine whether the growing use of antidepressants had an effect on European suicide rates, exploring the plausibility of competing explanations of associations with indicators such as adult per capita alcohol consumption, unemployment and divorce rates, and GDP. Our second aim was to examine temporal relationships, i.e. whether any reduction in the rate of suicide preceded any trend towards increased use of antidepressants as revealed by shorter and longer time-series of simultaneous antidepressant utilization and suicide data.

## Methods

### Sources of Data

This ecological and naturalistic study analyses correlations between datasets over a lengthy time period, covering 29 European countries including all 27 European Union Member States, with the exceptions of Malta and Cyprus, due to a lack of data on antidepressant utilisation in those countries. Data from Croatia, Iceland, Norway and Switzerland were also included.

Completed suicide data were obtained from the WHO Health for All European Mortality Database (WHO-MDB) [40]. This consisted of SDR for all cases of suicides (ICD10 codes X60–X84 and ICD9 codes E950–E959) for each available year for the period 1980–2009. We assumed the suicide recording procedures remained the same in the countries involved throughout the study period [41]. Population data and national unemployment rates were obtained from the WHO European Region Health For All Database (HFA-DB) [42]. Unemployment comprised all working age individuals out of work, currently available for work, or seeking work. GDP in US$ per capita was also obtained from the WHO HFA-DB [42]. Alcohol intake, defined as recorded adult (15+ years) per capita consumption of pure alcohol (APC) was obtained from the WHO Global Information System on Alcohol and Health (GISAH) [43]. The recorded crude divorce rate per 1000 population was obtained from OECD Social Indicators [44].

The defined daily dosage (DDD) of a drug for adults is determined by an independent scientific committee making use of the WHO Collaborating Centre for Drug Statistics Methodology [45]. Data on DDD per thousand individuals per day (DDD/1000/day) for antidepressants were used in the analysis. This data provides a rough estimate of use of these drugs and the proportion of the population receiving treatment with a particular antidepressant on a daily basis. Only antidepressants in class N06 of the Anatomical Therapeutic Chemical Classification System (ATC) were included in the analysis [45]. Other ATC drugs classes were excluded, such as lithium, bupropion, combination with antipsychotics and herbal remedies for depression such as St John’s Wort (*hypericum perforatum)*, because of a lack of data on consumption and/or consensus on average daily effective dose.

In order to maximise time series data on antidepressant use in each country over the period from 1990 to 2009, three different DDD/1000/day data sources were used. Total wholesale figures were obtained from IMS Health for the period 2004–2009 (1995–2009 for Portugal and 1996–2009 for Ireland) and OECD pharmacy sales data for the period 1990–2009 [46]. Data from national statistical offices and published literature for 1990–2009 were also used.

Country data from both the IMS and OECD were used wherever possible. For Bulgaria, Croatia, Ireland, Latvia, Poland, Romania and Switzerland only IMS Health data were available. Units of antidepressants sold each year in the IMS database were converted into kilogrammes of active ingredient in order to establish the total quantity of sold defined daily dose (DDDs), which were then divided by the country mid-year resident population, in order to obtain global DDD/1000/day. Using this procedure, we obtained units for total antidepressants, including tricyclic, atypical, SSRIs, Serotonin–norepinephrine reuptake inhibitors (SNRIs) and other antidepressants.

For Iceland, the Netherlands and Slovenia, OECD pharmacy sales DDD/1000/day data was the only available source. In addition to data from IMS and OECD, other DDD/1000/day data were obtained from the published literature, namely in the case of Austria [47], Hungary [48]–[50], and Italy [51] and directly from authors, as in the case of Denmark, Finland, Norway and Sweden [38], [52].

Where there was overlap in available information covering the same time period in any country, DDD/1000/day data were correlated to assess for consistency. After obtaining a very strong positive correlational analysis (r = .98) from these different sources, averaged DDD/1000/day were used in the analysis.

### Statistical Analyses

The final analyses were performed using 870 observations from 29 countries covering varying timeframes ranging from a maximum of 30 years (1980 to 2009) to just 4 years for antidepressant utilization in Slovenia. There was at least 20 years data for suicide rates in all countries, with 18 countries having data for all 30 years (Table 1). Rate of use for antidepressants and completed suicides in the first and last years for which data are available are presented, along with average annual trend data for each five year period covered. We did not use extrapolations based on available trends in consumption to estimate likely consumption of antidepressants for years where data were not available.

**Table 1:** ATC N06 antidepressant utilization (DDD per 1000 inhabitants per day) and utilization growth, and registered suicide standard death rate (SDR suicide, all) and rates variation, by year and country.

country	variables	period available	available years	first year	last year	Diff	last 5-years mean	growth %							
						last-first		period	per year	1980–1985	1985–1990	1990–1995	1995–2000	2000–2005	2005–2009
Austria	DDD/1000/day	1991–2009	19	9.1	56.54	47,44	49.78	521	27				115	*39*	*27*
	SDR suicide, all	1980–2009	30	25.09	12.8	−12,29	13.37	−49	−1.63	*5*	−*17*	−*7*	−13	−*16*	−*13*
Belgium	DDD/1000/day	1997–2009	13	29.3	67.76	38,46	61.43	131	10					*43*	*22*
	SDR suicide, all	1980–2006^1^	27	21.59	16.8	−4,79	17.19	−22	−.81	*2*	−*20*	*12*			
Bulgaria	DDD/1000/day	2004–2009	6	5.89	7.61	1,72	6.03	29	5						*74*
	SDR suicide, all	1980–2009	30	13.65	9.35	−4,30	10.01	−32	−1.07	*15*	−*10*	*10*	−3	−*29*	−*12*
Croatia	DDD/1000/day	2005–2009	5	16.45	22.9	6,45	19.56	39	8						*39*
	SDR suicide, all	1985–2009	25	22.23	14.96	−7,27	15.5	−33	−1.32		*4*	−*20*	*12*	−*18*	−*12*
Czech Republic	DDD/1000/day	1980–2009	30	2.7	35.88	33,18	30.93	1229	41	*0*	*15*	*74*	*80*	*157*	*44*
	SDR suicide, all	1980–2009	30	22.23	12.4	−9,83	12.42	−44	−1.47	−*8*	−*6*	−*16*	−*8*	−*6*	−*10*
Denmark	DDD/1000/day	1980–2009	30	8.96	77.6	68,64	68.5	766	26	*14*	−*17*	*118*	*87*	*71*	*32*
	SDR suicide, all	1980–2009	30	31.99	9.9	−22,09	10	−69	−2.30	−16	−*17*	−*29*	−*23*	−*17*	−*3*
Estonia	DDD/1000/day	1999–2009	11	5.2	13.14	7,94	13.41	153	14					*95*	*5*
	SDR suicide, all	1981–2009^2^	29	36.74	18.25	−18,49	17.32	−50	−1.72		−*13*	*49*	−*36*	−*28*	−*3*
Finland	DDD/1000/day	1980–2009	30	3.45	64.21	60,76	57.82	1761	59	*93*	*7*	*185*	*75*	*43*	*27*
	SDR suicide, all	1980–2009	30	25.24	18.26	−6,98	18.18	−28	−0.93	−*5*	*21*	−*10*	−18	−*18*	*4*
France	DDD/1000/day	1995–2009	15	28.9	49.26	20,36	49.47	70	5				*37*	*24*	*1*
	SDR suicide, all	1980–2008	29	18.99	14.96	−4,03	15.2	−21	−.72	*15*	−*13*	−*1*	−*10*	−*5*	
Germany	DDD/1000/day	1986–2009	24	6.1	39.95	33,85	33.34	555	23			*68*	*37*	*35*	*43*
	SDR suicide, all	1990–2009	20	15.47	9.51	–5,96	9.71	–39	–1.95			–10	–*16*	–*11*	–*9*
Greece	DDD/1000/day	1998–2009	12	11.6	49.14	37,54	43.71	324	27					*102*	*28*
	SDR suicide, all	1980–2009	30	3.2	3.02	–0,18	2.92	–6	–.20	*23*	–*18*	–*1*	–*1*	–*2*	–*3*
Hungary	DDD/1000/day	1990–2009^6^	20	3.77	25.45	21,68	24.19	575	29			60	*124*	*68*	*13*
	SDR suicide, all	1980–2009	30	44.54	21.79	–22,75	21.94	–51	–1.70	–*3*	–*12*	–*20*	–*4*	–*20*	–6
Iceland	DDD/1000/day	1989–2009	21	14.9	98.3	83,40	95.16	560	27			*98*	*114*	*34*	*4*
	SDR suicide, all	1980–2009	30	12.33	11.49	–0,84	11.48	–7	–.23	*22*	*7*	–*36*	*74*	–36	–*1*
Ireland	DDD/1000/day	1996–2009	14	17.96	55.51	37,55	49.91	209	15					*41*	22
	SDR suicide, all	1980–2009	30	7.69	11.61	3,92	10.21	51	1.70	*15*	*21*	*8*	*5*	–*11*	*8*
Italy	DDD/1000/day	1995–2009	15	8.88	36.39	27,51	33.3	310	21				*87*	*75*	*25*
	SDR suicide, all	1980–2008^3^	29	7.15	5.39	–1,76	5.24	–25	–.86	*10*	–*13*	2	–*13*		
Latvia	DDD/1000/day	2004–2009	6	4.15	6.14	1,99	5.59	48	8						*42*
	SDR suicide, all	1980–2009	30	32.6	20.7	–11,90	20.23	–37	–1.23	–*10*	–*11*	*57*	–*24*	–*27*	–*8*
Lithuania	DDD/1000/day	2002–2009	8	7.66	15.53	7,87	13.27	103	13						*54*
	SDR suicide, all	1981–2009^4^	29	35.16	31.47	–3,69	31.31	–10	–.34		–*23*	*76*	–*2*	–*21*	–*15*
Luxembourg	DDD/1000/day	2003–2009	7	36.2	47.93	11,73	44.56	32	5						*14*
	SDR suicide, all	1980–2009	30	12.88	10.8	–2,08	11.65	–16	–.53	*11*	17	–*15*	–*5*	–*27*	*11*
Netherlands	DDD/1000/day	2001–2009	9	31.4	40.1	8,70	39.42	28	3						*3*
	SDR suicide, all	1980–2009	30	10.56	8.52	–2,04	8.38	–19	–.63	*6*	*17*	–*1*	–*4*	*2*	–*5*
Norway	DDD/1000/day	1980–2009	30	7.99	54.19	46,20	53.03	578	19	*13*	*29*	*94*	*82*	*24*	*6*
	SDR suicide, all	1980–2009	30	12.67	11.45	–1,22	10.82	–10	–.33	12	*6*	–*19*	–*3*	–*5*	*2*
Poland	DDD/1000/day	2004–2009	6	10.59	16.66	6,07	14.09	57	10						44
	SDR suicide, all	1983–2009^5^	27	12.91	15.78	2,87	14.37	22	.81		–2	*6*	*3*	*0*	*5*
Portugal	DDD/1000/day	1995–2009	15	18.65	73.15	54,50	61.66	292	19				55	70	49
	SDR suicide, all	1980–2009	30	8.08	7.85	–0,23	7.51	–3	–.10	*24*	–*17*	–*12*	–*42*	*68*	*9*
Romania	DDD/1000/day	2004–2009	6	2.59	6.09	3,50	4.02	135	23						*183*
	SDR suicide, all	1989–2009	21	11.48	11.17	–0,31	11.12	–3	–.14			*34*	–*1*	–*8*	–*2*
Slovakia	DDD/1000/day	1996–2009	14	4.2	27.99	23,79	23.84	566	40					*130*	*42*
	SDR suicide, all	1986–2009	24	17.31	10.3	–7,01	10.13	–40	–1.67			–*14*	–6	–*11*	–*14*
Slovenia	DDD/1000/day	2006–2009	4	32.6	43.2	10,60	38.55	33	8						
	SDR suicide, all	1985–2009	25	33.64	18.71	–14,93	19.81	–44	–1.76		–*17*	–*2*	–*1*	–*19*	–*15*
Spain	DDD/1000/day	1992–2009	18	10.2	59.06	48,86	53.86	479	27				*76*	69	*24*
	SDR suicide, all	1980–2009	30	4.69	6.34	1,65	6.35	35	1.17	41	*8*	*2*	*1*	–*9*	–*4*
Sweden	DDD/1000/day	1980–2009	30	6.47	72.64	66,17	70.09	1023	34	*25*	*13*	*198*	*77*	*35*	*11*
	SDR suicide, all	1980–2009	30	19.04	12.33	–6,71	11.93	–35	–1.17	–*10*	–*8*	–*11*	–*18*	*6*	*0*
Switzerland	DDD/1000/day	2004–2009	6	40.87	49.08	8,21	46.62	20	3						*16*
	SDR suicide, all	1980–2009	30	24.94	12.5	–12,44	14.37	–50	–1.67	–*5*	–*15*	–*8*	–*7*	–*13*	–*16*
United	DDD/1000/day	1991–2009	19	10.4	61.93	51,53	55.01	495	26				*92*	*29*	*21*
Kingdom	SDR suicide, all	1980–2009	30	8.68	6.6	–2,08	6.47	–24	–.80	*1*	–*11*	–*8*	*0*	–*10*	*3*
Mean			15,28	13,69	43,91	40.33*	40,01		19,83						
			28,45	19,06	12,93	–6,16*	12,94		–0,81						

For some countries, data is missing for some years:

1 Belgium, years 2000 until 2003;

2 Estonia, years 1983 and 1984;

3 Italy, years 2004 and 2005;

4 Lithuania, years 1983 and 1984; and ^5^Poland, years 1997 and 1998 (SDR suicide, all);

6 Hungary, years 1992 and 1994 (DDD/1000/day).

* mean weighted by number of years of data available.

We examined the strength of the association between SDRs for suicide and the use of antidepressants measured in DDD/1000/day using Pearson’s correlation coefficient. We also used Pearson’s correlation coefficient to examine the direction and magnitude of associations between suicide SDRs or DDD/1000/day respectively and GDP, alcohol consumption, unemployment and divorce rates. We did not consider statistical significance because there are correlated measures within each individual country and independent measures in different countries. Therefore, p-values can only be obtained correctly through the use of a general linear mixed model, which we discuss below. This requires taking longitudinal co-variation between measures into account.

General linear mixed models (GLMM) combine the properties of linear mixed models which incorporate random effects and generalized linear models which contain non-normal data. The choice of a general linear mixed model (GLMM) allows for the correlation of observations and analysis of incomplete longitudinal data. It is a statistical method for modeling outcome measures as a function of fixed (population) effects, while simultaneously modeling individual subject parameters as random effects, and can accommodate time-dependent covariates as well as missing observations [53].

The GLMM is represented by
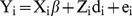
where Y_i_ is an n_i_×1 vector of n_i_ observations on the i-th subject; β is a p×1 vector of known, fixed, population parameters; X_i_ is an n_i_×p known, constant design matrix for the i-th subject; and d_i_ is a q×1 vector of unknown, random individual parameters. The random parameters are subject-specific but the vector size is the same from subject to subject; Z_i_ is an n_i_×q known, constant design matrix for the i-th subject corresponding to the random effects d_i_; and e_i_ is an n_i_×1 vector of random errors terms.

The GLMM is unstructured in relation to time frame, since the series of available years of data vary from country to country. The country is taken as the subject and a random effect, with DDD/1000/day, GDP, alcohol, unemployment and divorce as fixed effects, year as a repeating variable and suicide SDR as the dependent variable. The year of data observation was not considered a fixed effect because of its anticipated strong explanatory power for variations in suicide observations, which would prevent analysis of the role of other variables. This is also the reason we did not use time series.

In line with previous work looking at temporal patterns in fluoxetine prescribing and suicide rates in the US [54], co-variance analyses were performed according to first order auto-regressive (AR) models of aggregate time-series data to adjust for serial correlation in time series for each predictor (antidepressant use, GDP, alcohol consumption, and divorce and unemployment rates).

In order to assess the consistency of the GLMM results, we also performed a Poisson regression, an approach used in a number of previous studies [55]–[57]. In this case suicide SDR was the dependent variable, with DDD/1000/day as the predictor and a logarithm of base *n* of the number of years of available data per country, with an analysis of effects of Type III tests. To do this we cleaned the original database of years simultaneously without suicide SDR and DDD/1000/day, data were grouped by country, and the logarithm of base *n* time was created.

Finally, to compare the effect of changes in the use of antidepressants on suicide rates between two time periods, 1980–1994 and 1995–2009, an analysis was performed with an AR adjusted model, using DDD/1000/day as the independent variable and testing the interaction of DDD/1000/day and time period. Significance was set at p≤0.05 (two tailed). This demarcation of 1994, was chosen because it is the point where SSRIs started to become available and so, it was expected to mark an acceleration in the increase of DDD/1000/day over the subsequent 15 years.

Statistical analysis was done using SPSS software, version 17.0.

### Ethics Statement

These data are publicly accessible, with the exception of IMS Health, and are aggregated at the population-level. Individual-level information, for instance on individual patients, was unobtainable and therefore all data were analyzed anonymously without any privacy or confidentiality concerns. The Ethical Commission of the Faculdade de Ciências Médicas of Universidade Nova de Lisboa (medical institutional review board) where the two first authors are affiliated considers no review is needed if the data are anonymous and administrative.

## Results

### Trends in the Use of Antidepressants

On average there was 15 years of antidepressant utilisation data available in the 29 countries. There were marked differences: those countries with six or fewer years observations, with the exception of Switzerland, were all countries that have joined the EU since 2004 (Bulgaria, Latvia, Poland, Slovenia and Romania) or are in the process of joining (Croatia).

Data on the use of antidepressants are presented in Tables 1 and 2. Overall there has been an increase of 40.33 units DDD/1000/day in the study period, equal to the weighted-average difference between the first and last years of DDD/1000/day in each country, from an average of 13.69 to 43.91 in the first and last year of availability respectively. Our data indicates continuous growth in the use of antidepressants over time, albeit varying across countries, but with an average growth per annum of 19.83% in DDD/1000/per day. The lowest rates of annual growth of just 3% were seen in the Netherlands and Switzerland followed by Bulgaria, France and Luxembourg (all 5%), with the highest growth rate of 59% seen in Finland followed by the Czech Republic (41%), Slovakia (40%) and Sweden (34%).

**Table 2:** Pearson correlations between last 5-years means of DDD/1000/day and SDR suicide, million population and number of suicide deaths within quartiles of countries by years of SDR suicide and DDD/1000/d simultaneous data.

	all countries	countries by quartiles			
		19 or more years (P_>75_)	15 to 19 years (P_50–75_)	7 to 14 years (P_25–50_)	6 or less years (P_<25_)
Countries		Czech Republic, Denmark, Finland, Germany, Iceland, Norway, Sweden	Austria, Hungary,Italy, Portugal, Spain,UK	Estonia, France, Greece, Ireland, Lithuania, Luxembourg, Netherlands, Slovakia	Belgium, Bulgaria, Croatia, Latvia, Poland, Romania, Slovenia, Switzerland
DDD/1000/day and SDR Pearson correlation	–.41***	–.53***	–.46***	–.48***	.28
SDR – last 5 years available mean	12.94	12.08	10.15	14.21	15.33
DDD/1000/day – last 5 years available mean	40.01	58.41	46.30	34.70	27.82
Mid-year population –2009	514.1	117.7	196.9	105.2	94.3
Suicide deaths – last year available	60.903	14.563	16.508	14.857	14.975

*** p≤.001.

The latest available five year data indicate that the use of antidepressants varies markedly from just 4.02 DDD/1000 per day in Romania, 5.59 in Latvia and 6.03 in Bulgaria, to as much as 68.50 in Denmark, 70.09 in Sweden and 95.16 in Iceland. There was an average DDD/1000/per day of 40.01 across all countries.

### Suicide Trends

Suicide trends are presented in Tables 1 and 2. Over the study period, SDR rates for suicide decreased by a weighted average of 6.16, from an average SDR of 19.06 for the first year available to 12.93 in the last year available, notwithstanding variations in the years of data available in countries. The mean decrease in the SDR rate was 0.81%. There was little difference in the mean SDR rate for the last five years of data at 12.94.

Marked differences remained in suicide SDR rates across Europe, yet there was also a high degree of consistency in those countries with the lowest and highest rates of suicide over the study period. For the initial years of observation the highest rates were seen in Hungary (44.54), Estonia (36.74) and Lithuania (35.16), with the lowest rates seen in Greece (3.2), Spain (4.69) and Italy (7.15). For the last year of observation the highest SDR rates were seen in Lithuania (31.47), Hungary (21.79) and Latvia (20.7). Similarly the lowest rates were seen in Greece (3.02), Italy (5.39) and Spain (6.34).

Only Poland, Spain and Ireland had annual suicide rates higher in the last year compared with the initial year of observation. There was also little change in Greece, Iceland, Norway, Portugal and Romania but in all cases SDR rates were below the mean rate for all 29 countries. The highest rates of reduction were seen in Denmark, Estonia, Germany, Hungary, Slovakia, Slovenia and Switzerland.

### Correlation between Suicide and Utilization of Antidepressants

As Table 2 indicates, countries were grouped in quartiles by the availability of annual data on DDD/1000/day and SDR for suicides. A non-significant correlation was observable in countries with 6 or less years of both DDD and SDR data (r = .28; NS), they had the highest mean SDR suicide rate and the lowest DDD/1000/year for the last five year period, but covered less than a fifth of the population under study and less than a quarter of suicides in the last available year. For all other quartiles there is an inverse statistically significant correlation, with an increasing use of antidepressants and greater reductions in the suicide SDR.

**Figure 1:**
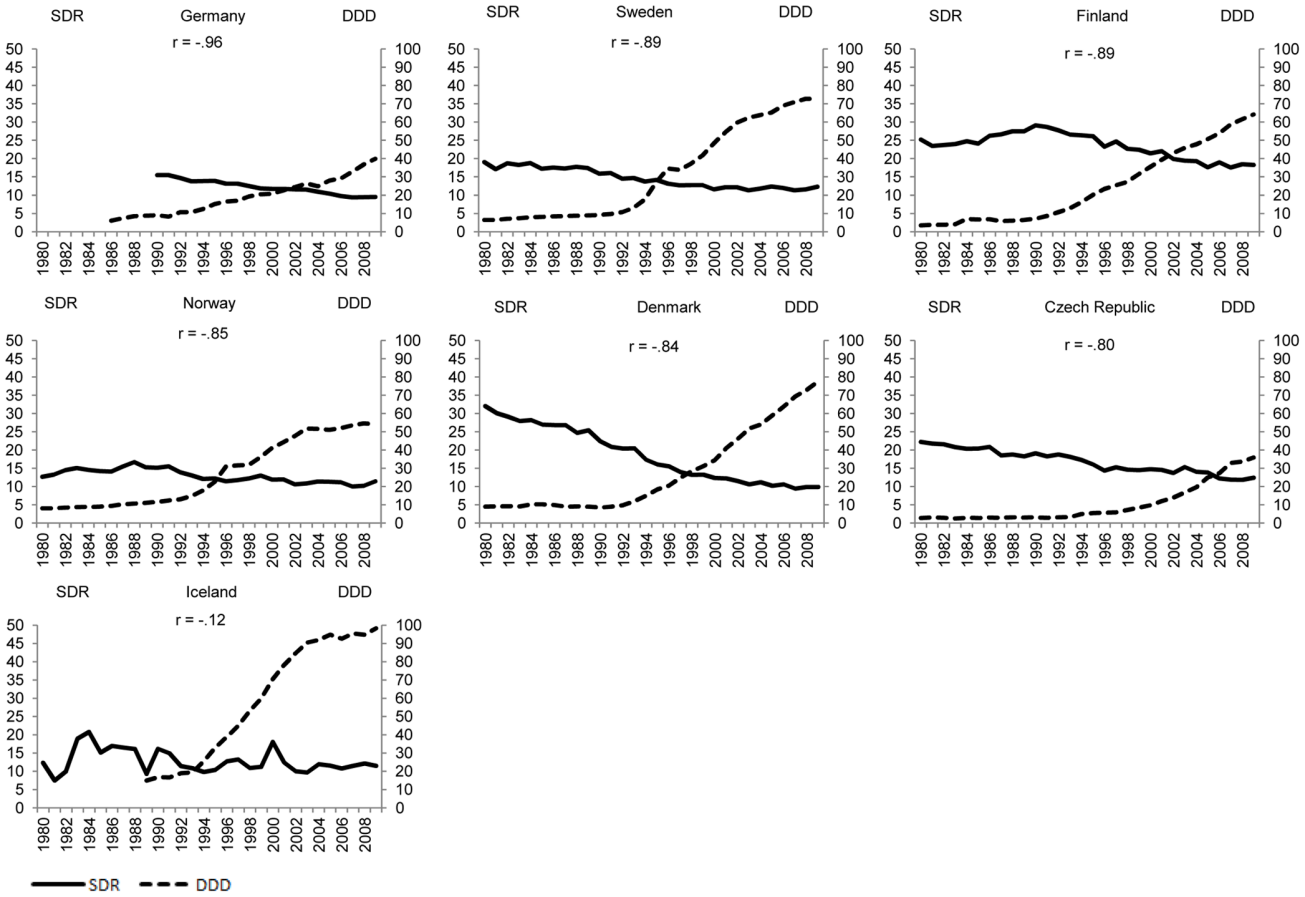
Suicide and use of antidepressants, by country, more than 19 years of simultaneous data (P_>75_).

**Figure 2:**
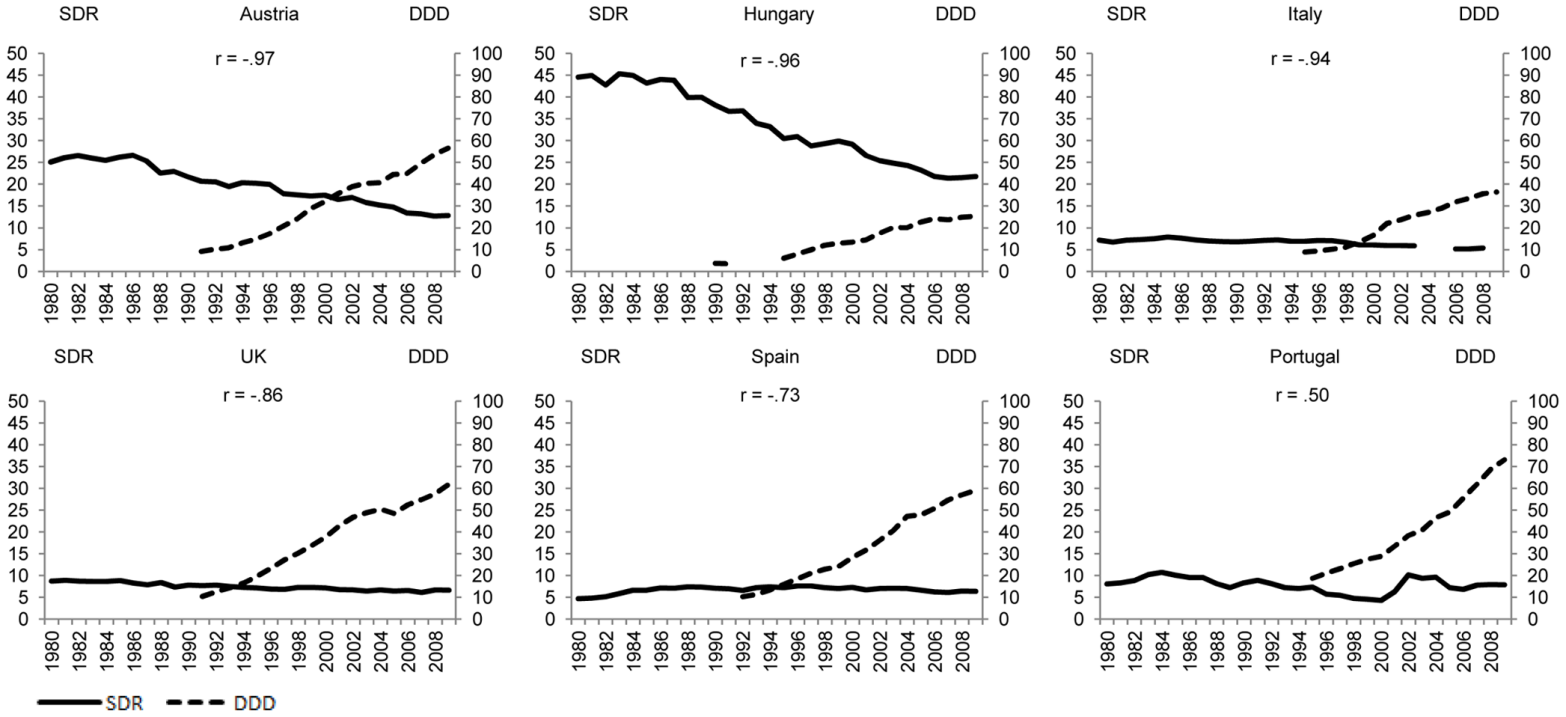
Suicide and use of antidepressants, by country, 15 to 19 years of simultaneous data (P_50–75_).

**Figure 3:**
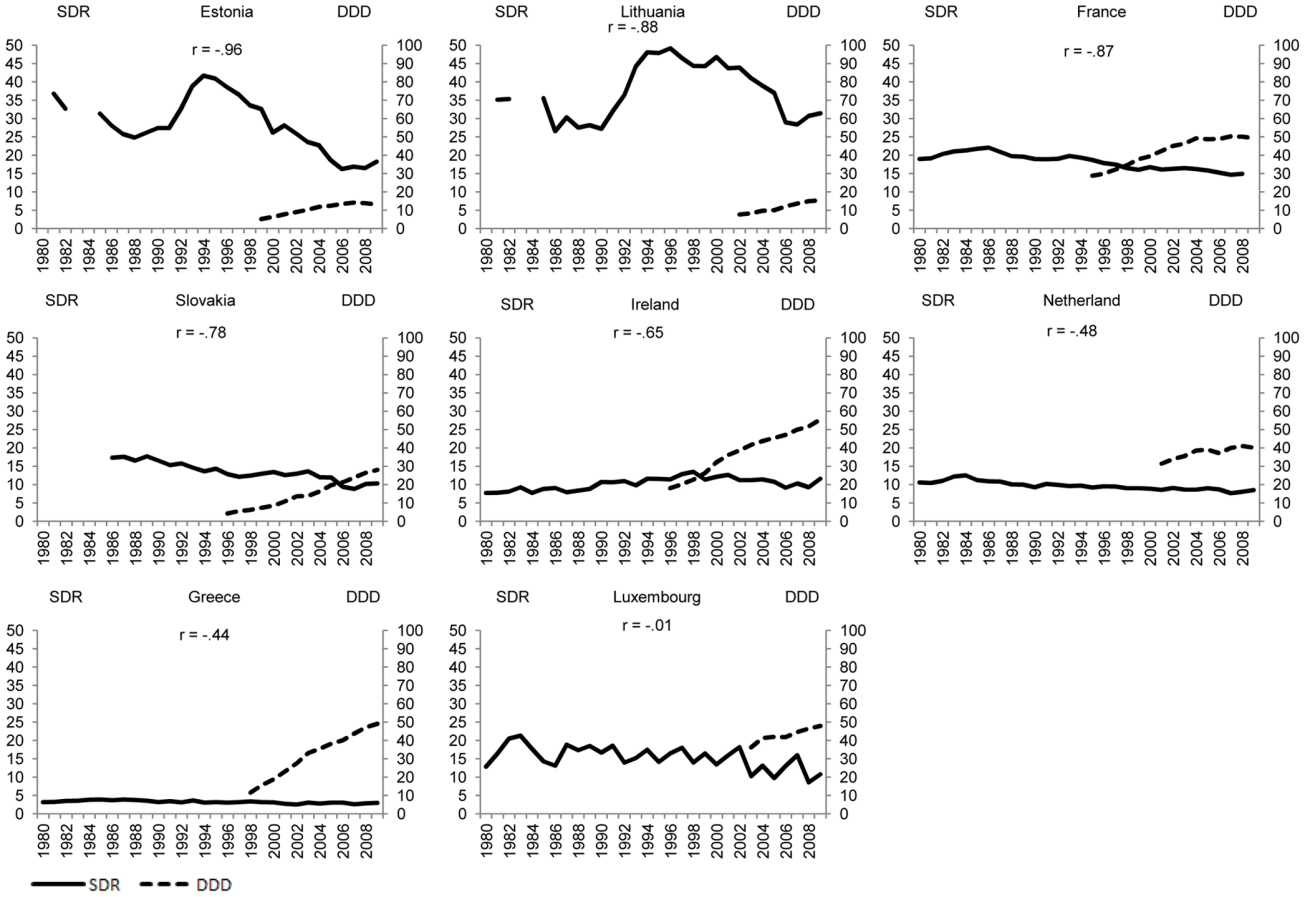
Suicide and use of antidepressants, by country, 7 to 14 years of simultaneous data (P_25–50_).

**Figure 4:**
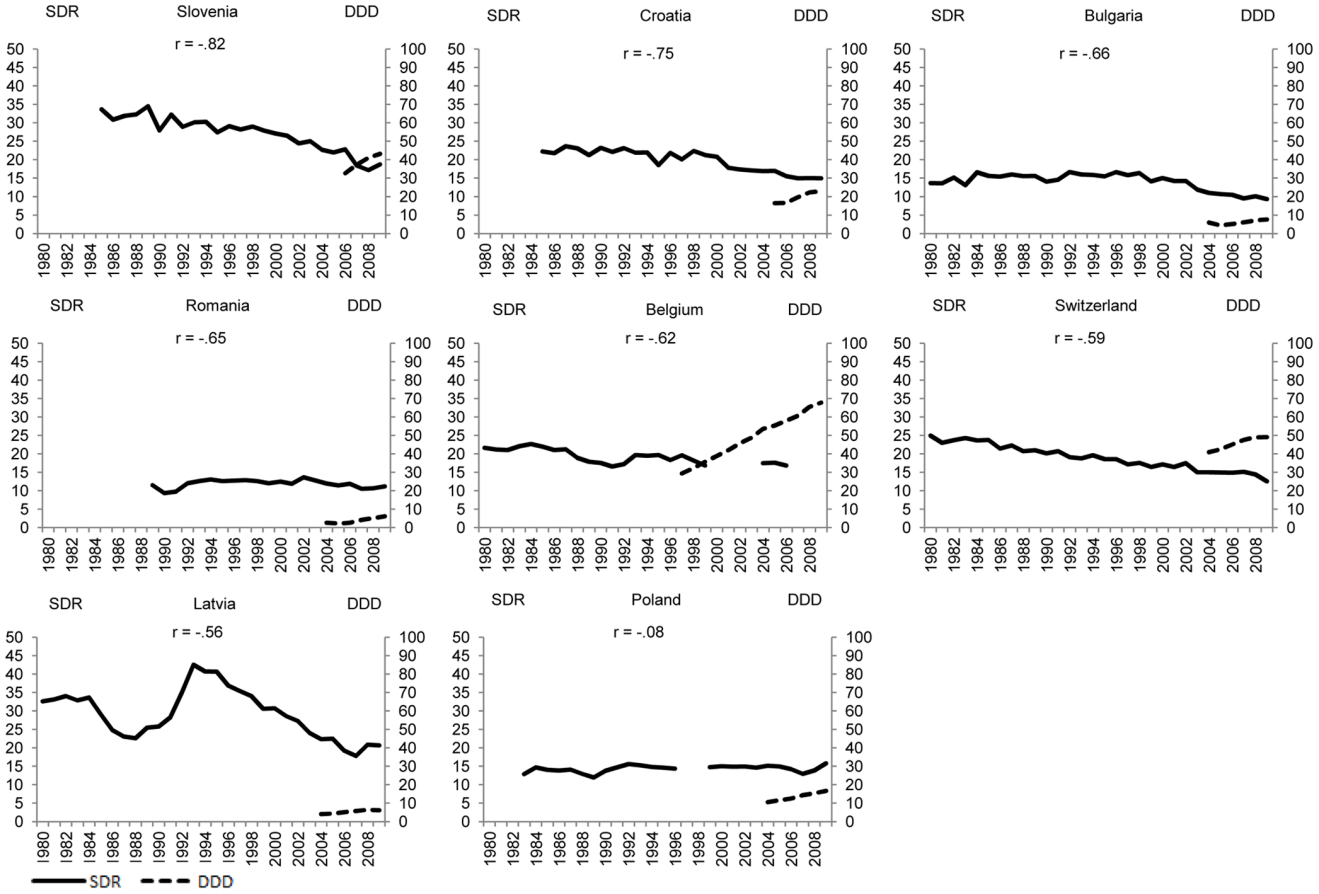
Suicide and use of antidepressants, by country, 6 or less years of simultaneous data (P_<25_).

Figures 1–4 plot suicide SDR and the use of antidepressants for the same country quartiles. In almost all countries, an increase of DDD/1000/day seems to correspond with a decrease in suicide SDR, although in countries where suicide rates are already low, antidepressants appear to have less impact. At first sight, the notable exceptions are Iceland (Figure 1), Portugal (Figure 2), and Luxembourg (Figure 3) whereas for the countries in Figure 4 the paramount characteristic is the lack of antidepressant utilization data.

### Association between Suicide and Use of Antidepressants with GDP, Alcohol, Unemployment and Divorce

We assessed the magnitude and direction of correlations in each individual country in an exploratory analysis with Pearson’s correlation coefficients. As Table 3 indicates, an inverse correlation was observable in all countries between SDR for suicide and DDD/1000/day, with the exception of Portugal. There was also an inverse correlation with GDP, with the exceptions of Ireland, Poland and Spain. As Table 4 indicates there is also a consistent direction and magnitude of correlation between DDD/1000/day and GDP in all 29 countries. No strong patterns are seen in either Tables 3 or 4 in respect of SDR and alcohol, unemployment or divorce.

**Table 3:** Magnitude of correlations between suicide SDR and antidepressant utilization and potentially confounding variables.

Country	DDD/1000/day	GDP	Alcohol	Unemployment	Divorce
Austria	–.97	–.94	–.05	–.66	–.92
Germany	–.96	–.77	.83	–.71	–.86
Hungary	–.96	–.86	.90	.18	.46
Estonia	–.96	–.78	–.88	.09	.72
Italy	–.94	–.81	.69	.66	–.81
Sweden	–.89	–.82	–.26	–.72	.03
Finland	–.89	–.66	–.54	–.04	–.09
Lithuania	–.88	–.67	–.33	.32	
France	–.87	–.86	.81	.02	–.54
UK	–.86	–.90	–.82	.77	.43
Norway	–.85	–.75	–.66	–.22	–.35
Denmark	–.84	–.89	.30	.65	.37
Slovenia	–.82	–.97	.27	–.22	–.48
Czech Republic	–.80	–.85	–.75	–.84	–.43
Slovakia	–.78	–.87	.47	–.26	–.79
Croatia	–.75	–.87	.34	–.10	
Spain	–.73	.12	–.67	.42	.30
Bulgaria	–.66	–.84	.01	.42	
Ireland	–.65	.38	.75	–.45	–.56
Romania	–.65	–.40	–.12	.65	
Belgium	–.62	–.78	.80	.59	–.57
Switzerland	–.59	–.93	.94	–.72	–.79
Latvia	–.56	–.68	.07	–.36	
Netherlands	–.48	–.86	.82	.89	.29
Greece	–.44	–.70	.57	–.54	–.52
Iceland	–.12	–.32	–.27	–.40	.26
Poland	–.08	.09	–.36	.56	–.31
Luxembourg	–.01	–.63	–.46	–.60	–.27
Portugal	.50	–.33	.46	.40	–.25

**Table 4:** Magnitude of correlations between DDD/1000/day and the other potentially confounding variables.

Country	GDP	Alcohol	Unemployment	Divorce
Austria	.80	–.91	.57	.81
Belgium	.92	–.79	–.65	.75
Bulgaria	.81	.26	–.17	
Croatia	.94	.99	–.91	
Czech Republic	.98	.80	.60	.37
Denmark	.91	–.44	–.86	–.03
Estonia	.92	.97	–.72	–.83
Finland	.89	.76	.21	.49
France	.71	–.60	–.84	.57
Germany	.88	–.63	.69	.65
Greece	.94	–.26	–.83	.90
Hungary	.92	–.47	–.18	.61
Iceland	.79	.92	–.04	–.52
Ireland	.92	.73	–.31	.77
Italy	.88	–.91	–.97	.94
Latvia	.97	.95	.29	
Lithuania	.95	.60	–.45	
Luxembourg	.90	–.48	.85	.13
Netherlands	.94	–.91	.39	–.73
Norway	.89	.81	.20	.48
Poland	.87	.78	–.85	.39
Portugal	.96	–.84	.64	.84
Romania	.81	.66	–.27	
Slovakia	.97	.54	–.43	.95
Slovenia	.80	–.95	–.78	1.00
Spain	.92	–.87	–.78	.88
Sweden	.85	.52	.54	–.21
Switzerland	.94	.46	–1.00	.20
UK	.90	.82	–.92	–.78

### Effects of Use of Antidepressants, GDP, Alcohol Consumption, Unemployment and Divorce on Suicide

The GLMM with DDD/1000/day as the only predictor of suicide rates (model 1) and with all independent variables as predictors (model 2) are displayed in Table 5. Model 1 revealed a significant effect of DDD/1000/per day (p = 0.002). In Model 2 DDD/1000/day still presents a significant effect on SDR suicide when adjusting for the other independent variables (p = 0.001). In this model an increase of one unit in DDD/1000/day, adjusted to the remnant independent variables, diminishes suicide SDR by 0.088 units. Divorce also appears to have a significant effect on SDR for suicide in this model (p = .007). An increase of one unit in the divorce rate, increases the SDR by 1.273.

**Table 5:** Model estimates of fixed-effects with SDR suicide rate as outcome.

	Regression coefficient	SE	T	p-value
**Model 1**				
DDD/1000/day	–.070	.022	–3.162	.002
**Model 2**				
DDD/1000/day	–.088	.026	–3.327	.001
GDP	.018	.026	.707	.480
Alcohol	.129	.159	.809	.419
Unemployment	–.015	.064	–.232	.816
Divorce	1.273	.473	2.692	.007

In Table 6 we present a Poisson distribution regression model where DDD/1000/day presents a significant, though only modest, effect on suicide SDR. The model has an adequate goodness of fit with a chi-square (27) = 1.118,83 inferior to the critical value for **α** = .001 and the Omnibus Test, a likelihood chi-squared test, is statistically significant.

**Table 6:** Poisson distribution regression with SDR suicide rate as outcome.

	Regression coefficient	SE	95% Wald confidence interval		p-value
			Lower	Upper	
DDD/1000/day	–.00018	.00004	–.00026	–.00010	<.001

We do not present a Poisson model with the other independent variables because of a high level of missing data after cleaning the database for empty cells: the number of observations was 870 in the GLMM but there are only 429 observations for suicide SDR and DDD/1000/year with Poisson regression. These would only be 311 observations when considering all independent variables.

### Effects of Antidepressant Utilization on Suicide Rates in Different Time Periods

Table 7 presents the results of our analysis comparing the effect of DDD/1000/day on suicide rates for two different time periods. This is a mixed model not structured within time, with countries with random effect, with DDD/1000/day and two periods (1980–1994 and 1995–2009) as fixed effects, with an interaction between DDD/1000/day and period, and year as a repeating factor. An effect modification between the two periods is observable since the interaction is very significant (p<0.001). In the period 1980–1994, the effect of DDD/1000/day on suicide SDR was –0.479, and –0.066 (–0.479+0.413) in the second time period. Thus there was a negative impact on suicide rates in both time periods, though this was much more pronounced for 1980–1994.

**Table 7:** Model estimates with total suicide rate as outcome and the interaction with period.

	Regression coefficient	SE	T	p-value
DDD/1000/day	–.479	.066	–7.232	<.001
Period	–6.991	.714	–9.791	<.001
DDD/1000/day×Period	.413	.064	6.460	<.001

## Discussion

### Main Results

Our analysis indicated that for 15 years of data on average for the 29 countries included in our study, the use of antidepressants increased on average by 19.83% per year. By the end of this time period for the whole study area there had been an average increase of 40.33 units of DDD/1000/day. Over a mean period of 28 years, the overall SDR for suicide decreased at a rate of 0.81% per year, corresponding to a reduction of 6.16 in the SDR rate for suicide. There was a strong inverse correlation between these trends.

How much of this increase of antidepressant use is needed to reduce the rate of suicide? A classical study from Sweden by Isacsson [38] covering the period from 1978 to 1996 found that suicide rates decreased consistent with a hypothesis that if the use of antidepressant medication increased five-fold, suicide rates would decrease by 25% assuming that depression treatment prevalence was approximately 1% and point prevalence of major depression was 5%. We have also already noted that in the large multi-national studies by Ludwig and Marcotte covering the period from 1980 to 2000 [31], [32], that an increase in SSRI sales of one pill per capita is associated with a decline in suicide rates of between 2.5% and 5% in different groups of countries around the world.

The total population of the 29 European countries in our study was 514.1 million in 2009. If 10 DDD/1000/day corresponds approximately to 1% of population treatment point prevalence [58], our data suggests that there could have been an increase in treatment for depression of 4% of this population correlating with a saving of 31.670 deaths by suicide in the last year covered, equivalent to 650 people treated for each life saved, per year.

Portugal is the only country where there is a positive correlation between DDD/1000/day and suicide SDR, considering the actual large utilization of antidepressants. This can possibly be explainable by the lack of precision of suicide register and over-estimation of undetermined violent deaths concealing suicides [41], [59]. The small populations of both Luxembourg and Iceland and therefore the small numbers of suicides recorded, probably accounts for the lack of any relationship with high antidepressant utilization. In other countries, such as Greece or Ireland, there was a sharp increase in DDD/1000/day and little change in suicide was apparent though a clear negative correlation was present. Perhaps in Greece, with an already very low suicide rate along the period a ceiling effect is present whereas in Ireland suicide is more frequent in young and middle aged men, who typically present an unfavourable help-seeking behaviour [60].

Another key result was the demonstration of how the impact of antidepressant utilization on suicide changes as more annual information becomes available on both DDD/1000/day and SDR suicide: a longer series of data means a stronger correlation between lower SDR for suicide and higher DDD/1000/day. This suggest that those countries in this study with less than 6-years of both types of data that did not show a significant inverse correlation between these indicators may well in future demonstrate this finding as more data becomes available. Previous published studies with negative results should be analysed in view of this contingency.

A third important result was that two different regression statistics confirmed that DDD/1000/day is an explanatory factor for suicide SDR, notwithstanding that the Poisson regression meant reducing analysed observations from 870 to 426, therefore reducing accuracy in relation to the GLMM. Though we cannot assume a causal effect, when adjusting for other independent variables, adequate GLMM modelling makes DDD/1000/day an explanatory factor for changes in the suicide SDR: a one unit increase of DDD/1000/day seems to diminish the suicide SDR by 0.088 units.

A clear covariance is observable, at the country level, between potentially explanatory suicide factors such as antidepressant utilisation, GDP, alcohol consumption, unemployment and divorce, suggesting a differential impact on countries and implying GLMM and Poisson regressions were appropriate models for estimation.

The fourth key result was the demonstration that DDD/1000/day had an effect on SDR for suicide both between 1980 and 1994 and in the subsequent 15 years. With increasing DDD/1000/year, suicide-related SDR was expected to decrease. However, this decreasing trend decelerates over time: the analysis suggests that in the first period, where in most cases high rates of suicide SDRs were seen, less of an increase in the use of antidepressants would be necessary to reduce suicide SDR whereas in the subsequent period, when suicide SDRs had become lower in most countries, a much higher rate of antidepressant utilization would be necessary to further reduce suicide SDR. Suicide-related SDR continued to decline in the second 15-year period, albeit at a much reduced rate. More importantly, nevertheless, it suggests that antidepressant utilization DDD/1000/day increase had an important effect in suicide SDR from the start, when suicide SDR started to lower in Europe. This rebuffs most criticisms and scepticism on observable antidepressant effects on suicide decrease, usually stating that suicide had already started to decrease before antidepressant utilization exploded, in the nineties, therefore denying earlier generation antidepressant effects.

### Previous Studies

This study is similar to those of Ludwig and Marcotte [31], [32] since the observation unit is the country. Nevertheless, it presents several characteristics that might be seen as advantageous in relation to the generalisability of results. First, it draws on a more homogeneous set of countries, albeit with some substantial differences in GDP, infrastructures and historical development of countries that had formerly been part of the Soviet bloc prior to 1990. Second, it covers more consecutive years of effects versus each 5 year calculation (average 15 years against 10 years, since in Ludwig and Marcotte’s studies for 1980–1990 SSRI pills are extrapolated), the almost global extension of antidepressants (and not only of SSRIs), the use of DDD/1000/day, which is a measure of antidepressant utilization independent of national regulations, costs and commercial specificities, allowing for comparisons, and that can be related to utilization needs and treated prevalence. As previously explained, one DDD may be sufficient for one person-day of adequate treatment, and 10 DDDs per 1000 inhabitants per day is therefore considered to represent approximately a 1% point treated prevalence [58] though we cannot assume users take medication as prescribed.

This study has also many advantages over single national studies, though it does not substitute for them: it controls for the variability of factors that affect suicide rates at the country level and it has more explanatory power on the role of antidepressants on suicide rates because of the number of observations involved. We also believe it purports more power than reviews and meta-analysis produced so far because of its consistency: close geographic and socio-political context in the last 10 years, use of available annual data series, inclusion of all families of antidepressants and use of DDD/1000/day.

In fact, single studies and reviews [29] have used several definitions of antidepressant utilization, including costs, number of packages or pills sold, number of prescriptions issued, defined daily dosage (DDD) and defined daily doses per thousand individuals per day (DDD/1000/day). These discrepancies hinder comparability and introduce probable sources of bias because drug costs, pills dosages, quantities of pills per package and prescriptions, might oscillate longitudinally because of external, regulatory and commercial reasons. Using DDD/1000/day represents a stable variable for the estimation of the exposure to drugs and the proportion of the population that may receive treatment with a particular drug on a daily basis [45].

Another source of bias in some previous studies has been associated with a major focus on SSRIs, and not on the analysis of the use of the whole class of antidepressants, including SNRIs, atypicals and tryciclics. Patterns in the use of these drugs might vary considerably across countries.

Moreover, these studies present substantial differences in the periods of time that are analysed, both for antidepressant use and recorded suicides, from as low as 2 years to as high as 30 years for antidepressant use utilization series, and a similar but slightly better pattern for suicide time series. It is likely that these variations will have had an impact on results, as well as making meaningful comparison difficult.

The extent to which data series are available across countries for the same time periods can influence correlation results strikingly. This is quite important to assess previous and future studies: results are far more reliable when longer yearly time series are present. Our study presents an average of 15 years of both annual suicide and antidepressant utilization data, the largest figure to our knowledge, albeit hampered by inconsistent time series data for antidepressant utilization across countries. There is also considerable variability of statistical procedures within studies published so far, which further complicates comparing results. Most studies present differing correlations, linear regressions, Poisson regressions and time series; we avoid this problem in our multi-country analysis.

### Ecological Design Considerations

Because this is an ecological study, we emphasise that we cannot depict causal links and therefore these results must be interpreted with great caution. Nevertheless, as argued earlier, we believe that there is a case for this study design because there is a need to validate the effectiveness and potential cost effectiveness of antidepressants as an intervention for suicide prevention. As suicide is a comparatively rare event, it would be very difficult to study in a controlled trial, not to mention any of the potential ethical concerns that might arise in trial design [38], [39]. Trials are also unlikely to be of a sufficiently long timeframe for adequate analysis of impacts on suicide, where data covering many years is required. Thus ecological studies still have a place in the evaluation of some interventions, namely public health interventions. As in this case, conclusions drawn do not uncover causal links but must be taken in view of knowledge available, face validity and plain common sense.

The debate on suicide-antidepressant trends at the public health level might be seen as disproportionate since the methodological and interpretation problems that arise are present in all ecological studies exploring any kind of correlations. For instance, there have been positive developments in mental health services policy, delivery and provision in Europe in recent decades [61] but the impact of mental health services provision on suicide rates assessed through ecological studies has produced mixed results [47], [48], [62]–[68]. Nevertheless, it would hardly be arguable not to implement good practices and optimise mental health services when resources are available.

### Confounding Effects

The association between alcohol consumption, unemployment, and divorce and suicide was inconsistent across the different countries in our analysis. How can an inverse correlation between these variables and the suicide rate in some countries be interpreted, when a positive correlation was expected?

Considering alcohol consumption, even though patterns of alcohol use differ from culture to culture, it is known that alcohol abuse can contribute to an increased risk of suicidal behavior [24], [69]. Therefore, it would be expected that a reduction in suicide would be accompanied by a reduction in alcohol intake [70]. Notwithstanding, in Hungary, between 1990 and 1998 alcohol sales increased by 25% and suicide rates dropped by 20% [48] and in a Hungarian suicide prevention project, the intervention region had a higher alcohol-related death rate both before and after the program compared with the control region. Moreover, there was a decrease in alcohol-related deaths over time in both regions, and the expected improvement in the intervention region was not confirmed [71].

Also, in a previous study performed in 1980–1982 in Portugal, the inverse association between suicide rates and alcohol cirrhosis deaths had a distinctive regional distribution: whereas in the north, alcohol uptake and cirrhosis death was greater than in the south, suicide rates were much greater in the south [22], suggesting that alcohol addiction is on many occasions either a depression equivalent or a self-medication strategy [24]. Overall, cultural differences are also important in explaining variation in the associations across countries.

### Explanations for Increased Prescribing of Antidepressants

Along with the perceptions of newer antidepressants of being safer and easier clinically to manage, the past 30 years has been characterized by depression awareness campaigns and more extensive medical training concerning depression and suicide [48]. Training for primary care and other medical personnel concerning depression and suicide risk management has been a core component of many suicide prevention programmes in Europe since the implementation of the Gotland study [7], [72]–[75]. Moreover, there is increased awareness of the extent of the impact of poor mental health and the increased need for treatment and support in Europe [76], [77], varying from country to country [78], that may also contribute to this increase in antidepressant prescribing. Finally, there has been some increased funding for mental health systems during the observation period. This may have helped make antidepressants, along with other treatments for depression more accessible [79].

### Limitations

There are a number of limitations to this analysis. Utilisation is only a proxy for rates of what occurs at the individual patient level: we do not know if people take the medication they obtain, or if they are taking less or more than the standard DDD. The real rates of treatment of depression in Europe could conceivably be lower than the high DDD/1000/day would suggest, taking in account the multiple indications of antidepressants, frequent use of higher dosages than the DDD, non-compliance, and co-therapy with a second antidepressant [38]. Nor do we know the proportion of individuals taking these medications that complete suicide. In addition, we do not know the gender and age distribution of antidepressant use, and so have not attempted to look at the impacts of antidepressants on suicide rates by age or gender. We also do not know the distribution of utilisation in relation to severity of depression and anxiety disorders although some surveys in Europe suggest the gap is greater in the lower end of severity, and a recent meta-analysis suggests the value of antidepressants for light and moderate as well as severe depression [80].

It should also be acknowledged that antidepressants are prescribed for other mental health problems in addition to depression (e.g. anxiety disorders, anorexia and bulimia nervosa, ADHD), as well as for physical health problems (e.g. migraine headaches, fibromyalgia, chronic pain) [81]–[83]. We also know that poor physical health can be a risk factor for suicide [84].

Our analysis is also limited by focusing only on completed suicides, but a proportion of undetermined deaths will also be due to suicide; potentially including these data in our analysis might impact on findings, particularly in countries such as Portugal and Eastern Europe countries where undetermined deaths are considerable. Future analysis should consider ‘probable suicide’ i.e. the sum of registered suicides and undetermined violent deaths. Another limitation of our analysis is a lack of data on the use of psychological therapies, alongside or as an alternative to the use of antidepressants in treating depression and related disorders, and therefore potentially contributing to the prevention of suicides. Many of these limitations can only addressed through improvements in epidemiological datasets, including more information about treatment pathways; surveys might also be considered to better obtain data on the use of antidepressants and other medications, as well as other psychosocial therapies among specific population sub-groups.

Nonetheless, despite these limitations and our caution over the interpretation of our findings, the outcomes of the present study underline the need to better optimise the appropriate use of antidepressants as part of routine care, given that many people who may benefit from their use do not receive them, while conversely other individuals are inappropriately taking such medications. Whether research projects, such as OSPI-Europe that seek to foster a better quality of care, starting at the primary care level, focusing on improved awareness of depression and risk of suicide, appropriate antidepressant and other treatment prescribing and monitoring, might produce such an effect will require empirical demonstration.
